# rSjp40 inhibits activated hepatic stellate cells by promoting nuclear translocation of YB1 and inducing BMP-7/Smad1/5/8 pathway

**DOI:** 10.1186/s13071-019-3539-z

**Published:** 2019-05-31

**Authors:** Liuting Chen, Qi Zhou, Ertao Liu, Jiali Zhang, Lian Duan, Dandan Zhu, Jinling Chen, Yinong Duan

**Affiliations:** 1 Department of Pathogen Biology, School of Medicine, Nantong University, Nantong, 226001 Jiangsu People’s Republic of China; 2 Department of Orthopedics, Nantong Fourth People’s Hospital, Nantong, 226005 Jiangsu People’s Republic of China; 3 Department of Medical Informatics, School of Medicine, Nantong University, Nantong, 226001 Jiangsu People’s Republic of China

**Keywords:** Hepatic stellate cell (HSC), Bone morphogenic protein-7 (BMP-7), Y-Box protein-1 (YB1), Fibrosis, Signal transduction, *Schistosoma japonicum*

## Abstract

**Background:**

Activation of hepatic stellate cells is the dominant pathogenic event during the process of liver fibrosis. Bone morphogenic protein (BMP)-7 has recently been identified as an anti-fibrotic factor and leads to phosphorylation of Smad1/5/8 in activated hepatic stellate cells. Its expression can be upregulated by the transcriptional activator, Y-Box protein-1 (YB1). Previous studies have found that the recombinant *Schistosoma japonicum* protein p40 (rSjp40) can inhibit the activation of hepatic stellate cells, and based on this evidence we attempted to investigate whether or not BMP-7 is involved in rSjp40’s inhibition.

**Methods:**

A human hepatic stellate cell line, the LX-2 cell line, was cultured and treated with rSjp40. The role of BMP-7 was analyzed by Western blot.

**Results:**

Our findings testified that knockdown of BMP-7 impaired rSjp40-induced downregulation of α-SMA and phosphorylation of Smad1/5/8 in LX-2 cells. Furthermore, rSjp40 upregulated expression of BMP-7 at both mRNA and protein levels depending on YB1. Interestingly, YB1 was translocated from the cytoplasm to the nucleus upon treatment of rSjp40.

**Conclusions:**

These results suggest that rSjp40 inhibits the activation of hepatic stellate cells by promoting nuclear translocation of YB1 and inducing BMP-7/Smad1/5/8 pathway, which provide a new clue to guide ongoing research into the anti-fibrosis of rSjp40.

## Background

Liver fibrosis is a pathological process due to most chronic liver injuries like viral hepatitis, alcohol abuse, metabolic disease, and parasite infection. Both quantitative and qualitative changes in the composition of liver extracellular matrix (ECM) occur during the development of liver fibrosis. The total ECM increases 3–5-fold, accompanied by the shift in the type of ECM from the normal low density basement membrane-like matrix to interstitial type matrix containing types I and III collagens [1]. Accordingly, activated hepatic stellate cells (HSCs), characterized by the appearance of α-smooth muscle actin (α-SMA), are the main ECM-producing cells [2]. Of note, HSC activation is particularly promoted by profibrogenic cytokines, with transforming growth factor-β1 (TGF-β1) being the most potent [3]. The signaling of TGF-β1 leads to the phosphorylation of receptor-specific Smad (R-Smad) proteins, Smad2 and Smad3. Then R-Smads together with a common mediator Smad4 translocate to the nucleus, where they enhance transcription of collagen genes, inhibit synthesis of ECM degradation enzymes and upregulate expression of tissue inhibitors of metalloproteinases to promote liver fibrosis [4].

Bone morphogenic protein (BMP)-7, a member of the TGF-β superfamily, has been reported to counteract some of the profibrogenic actions of TGF-β1 [5]. It initiates the signal transduction cascade by allowing phosphorylation of Smad1/5/8. Smad1/5/8 forms a complex *via* binding to Smad4, thus, being translocated to the nucleus to suppress the accumulation of Smad3 by TGF-β1 stimulation [6, 7]. In liver fibrosis, the involvement of BMP-7 has only recently been suggested. Oral administration of recombinant adeno-associated virus carrying BMP-7 in mice led to an increased circulating BMP-7 concentration and resulted in amelioration of CCl_4_-induced liver fibrosis and HSC activation [8]. Injection of adenoviral BMP-7 promoted the reversion of liver fibrosis/cirrhosis in the CCl_4_-induced cirrhotic hamster model [9]. And in other animal models like thioacetamide- or repeated intraperitoneal injection of porcine serum-induced liver fibrosis in rats, ectopic expression of BMP-7 can also inhibit the fibrogenic progress [10, 11]. Furthermore, in the *Schistosoma japonicum*-induced liver fibrosis mouse model, administration of recombinant BMP-7 intraperitoneally significantly decreased the degree of collagen deposition and the expression of α-SMA in the liver, measured by Masson’s staining and α-SMA staining [12].

Y-Box protein-1 (YB1) is a DNA- and RNA-binding protein and identified as a transcriptional activator of BMP-7, which binds to the proximal region of BMP-7 promoter (−192 to +3 bp). It is reported that knockdown or knockout of YB1 can reduce BMP-7 expression. In the high glucose condition, YB1 was translocated to the nucleus to increase the BMP-7 promoter activity [13, 14].

Schistosomiasis is an infectious disease that affected 207.7 million people worldwide in 2016, according to the latest Global Health Observatory (GHO) data. Most evidence suggests that schistosome eggs induce the morbidity caused by schistosome infection [15]. However, studies in recent years have found that schistosome eggs can restrain the activation of HSCs and induce the downregulation of fibrogenesis [16, 17]. In our laboratory, we also found that soluble egg antigens (SEA) from *Schistosoma japonicum* can induce the suppression of activated human stellate cell line, LX-2 cells and primary mouse HSCs through the TGF-β1 and PPARγ signaling pathways [18]. SEA is a complex mixture which is isolated from schistosome eggs. And Sjp40 is a major component of SEA from *Schistosoma japonicum* [19]. In previous research, we have expressed and purified the recombinant Sjp40 protein (rSjp40), and the preceding studies have demonstrated that rSjp40 potently inhibits the activation of HSCs to exert its anti-fibrotic effect [20–22].

In this study, we identified that BMP-7 is involved in rSjp40’s inhibition on HSC activation. Knockdown of BMP-7 inhibited rSjp40-induced phosphorylation of Smad1/5/8 and downregulation of α-SMA. Mechanistically, YB1 was translocated to the nucleus and promoted BMP-7 expression after rSjp40 treatment. Our findings reveal a previously unidentified target and provide insight into the mechanisms of rSjp40’s inhibition on HSC activation.

## Methods

### Reagents

rSjp40 protein was obtained as previously described [23]. Rabbit pAbs against GAPDH (Goodhere, Hangzhou, China) or YB1 (Abcam, Cambridge, UK), mouse mAbs against BMP-7 or α-SMA (Santa Cruz Biotechnology, Dallas, TX, USA), horseradish peroxidase (HRP)-conjugated anti-rabbit IgG (Biosharp, Hefei, China), HRP-conjugated anti-mouse (Santa Cruz Biotechnology, Dallas, TX, USA) were purchased from the indicated companies.

### Cell culture

LX-2 cells were grown in DMEM (Gibco, Waltham, MA, USA) supplemented with 10% FBS (Thermo Fisher Scientific, Waltham, MA, USA).

### RNA interference

Small interfering RNAs (siRNAs) corresponding to the target sequences were purchased from GenePharma (Shanghai, China). The following sequences were targeted for human BMP-7 or YB1 cDNA: BMP-7-siRNA forward (5′-GCC UGC AAG AUA GCC AUU UTT-3′) and reverse (5′-AAA UGG CUA UCU UGC AGG CTT-3′); YB1-siRNA forward (5′-GCC AAU AGA AGC UAG GGA UTT-3′) and reverse (5′-AUC CCU AGC UUC UAU UGG CTT-3′). A negative control siRNA (Con-siRNA) was used in parallel. LX-2 cells were seeded on 6-well plates and transfected with the indicated siRNA (300 pmol) on the following day by Lipofectamine 2000 (Invitrogen, Waltham, MA, USA) according to the manufacturer’s instructions. Forty-eight hours later, cells were treated with or without rSjp40 (5 μg/ml) for another 48 h.

### Western blot

Cells were harvested and resuspended in RIPA cell lysis buffer [200 mM Tris-HCl (pH 7.5), 150 mM NaCl, 1 mM EDTA, 1% Triton X-100] with 1% PMSF (Biosharp, Hefei, China) and phosphatase inhibitor complex III (1 mM) (Sangon Biotech, Shanghai, China). Samples were separated by 10% SDS-PAGE and then transferred from the gels to polyvinylidene difluoride (PVDF) membranes. After blocked in 5% nonfat milk, the membranes were incubated with the indicated primary and secondary antibodies. Protein bands were visualized with ECL system (Biorad, Berkeley, CA, USA).

### Reverse transcription-quantitative real time PCR

Total RNA extraction, reverse transcription and quantitative real time PCR analysis were performed as previously described [24]. Gene-specific primer sequences were as follows: *GAPDH*, forward (5′-GAC AAG CTT CCC GTT CTC AG-3′) and reverse (5′-GAG TCA ACG GAT TTG GTC GT-3′); *BMP-7*, forward (5′-GGC TGG CAG GAC TGG ATC AT-3′) and reverse (5′-ACC AGC GTC TGC ACG ATG GC-3′).

### Isolation of cytoplasmic and nuclear fraction

LX-2 cells left untreated or treated with rSjp40 (5 μg/ml) for 48 h were harvested and separated using the Nuclear and Cytoplasmic Protein Extraction kit (ApplyGen, Beijing, China) according to the manufacturer’s instructions.

### Immunofluorescence analysis

LX-2 cells were seeded on 24-well plates and left untreated or treated with rSjp40 (5 μg/ml) on the following day. Forty-eight hours after treatment, cells were fixed by 4% paraformaldehyde and permeabilized by 0.1% Triton X-100. Then cells were incubated with the primary Ab against YB1 at 4 °C overnight, followed by AlexaFluor 594 secondary Ab (Molecular Probes, Waltham, MA, USA) for 2 h at room temperature. The cells were also stained with Hoechst 33342 (Sigma-Aldrich, Saint Louis, MO, USA) for 15 min and observed by fluorescent microscopy.

### Statistical analysis

All experiments were analyzed by the Student’s t-test. A *P-*value < 0.05 was considered significant.

## Results

### Knockdown of BMP-7 impairs rSjp40’s inhibition on HSC activation

**Fig. 1:**
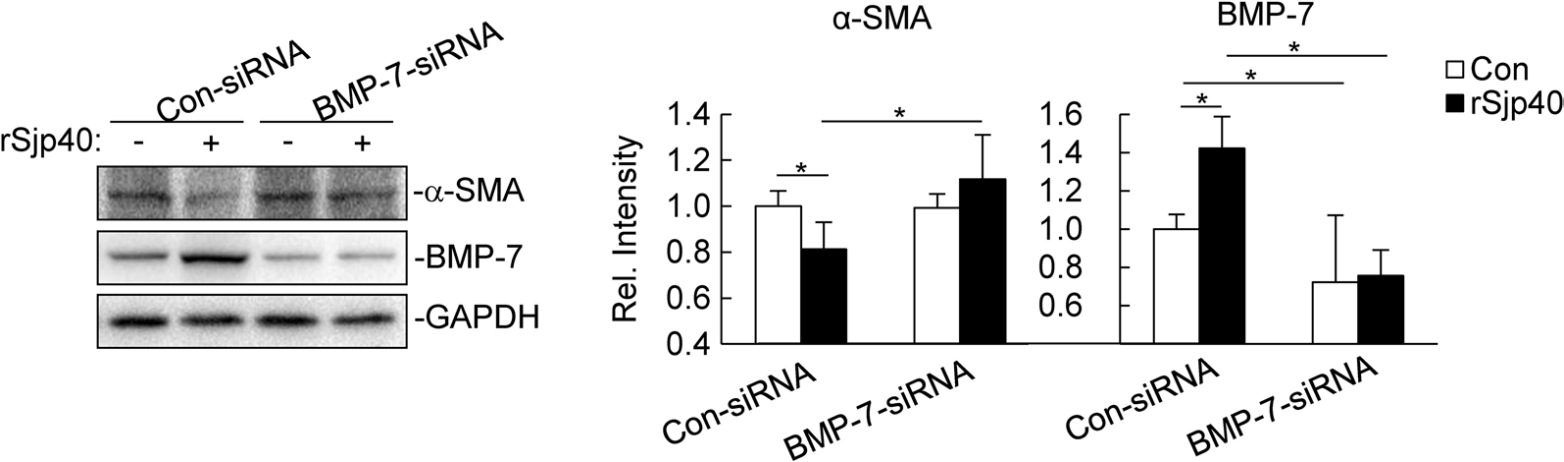
Knockdown of BMP-7 impairs rSjp40’s inhibition on HSC activation. LX-2 cells (2–3 × 10^5^) were transfected with Con-siRNA or BMP-7-siRNA (300 pmol each). Forty-eight hours after transfection, cells were left untreated or treated with rSjp40 (5 μg/ml) for 48 hours before immunoblot analysis was performed with the indicated Abs. The bar graphs show the relative intensities of the bands, which were quantitated by densitometry using Image Lab, normalizing to GAPDH levels. Data are represented as the mean ± SD (*n* = 3). *P < 0.05

To investigate whether BMP-7 is involved in rSjp40’s inhibition on HSC activation, we used BMP-7 specific siRNA to knockdown the expression of BMP-7 in LX-2 cells (t-test: *t*_(4)_ = 3.081, *P* = 0.0185). As shown in Fig. 1, the protein level of α-SMA was decreased after rSjp40 treatment in con-siRNA group (t-test: *t*_(4)_ = 2.409, *P* = 0.0368), which was consistent with our previous results [20–22]. However, knockdown of BMP-7 inhibited rSjp40-induced downregulation of α-SMA (t-test: *t*_(4)_ = 2.353, *P* = 0.0391). These results suggest that BMP-7 plays an important role in rSjp40’s inhibition on HSC activation.

### rSjp40 inhibits activation of HSCs partially *via* BMP-7/Smad1/5/8-dependent mechanism

**Fig. 2:**
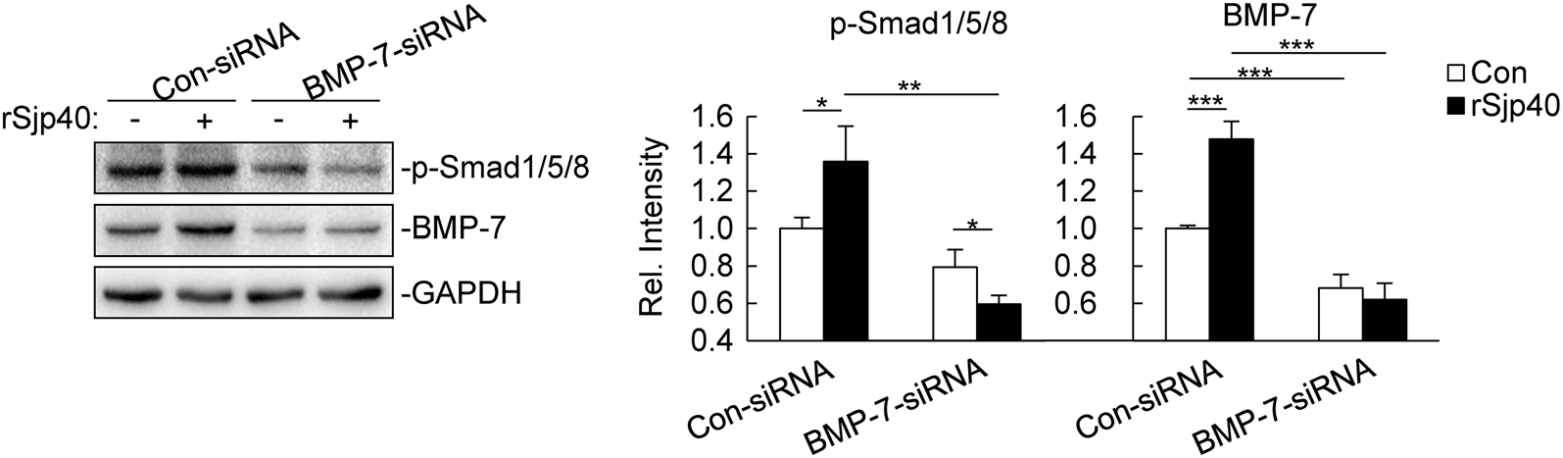
rSjp40 inhibits activation of HSCs *via* BMP-7/Smad1/5/8-dependent mechanism. LX-2 cells (2–3 × 10^5^) were transfected with Con-siRNA or BMP-7-siRNA (300 pmol each). Forty-eight hours after transfection, cells were left untreated or treated with rSjp40 (5 μg/ml) for 48 hours before immunoblot analysis was performed with the indicated Abs. The bar graphs show the relative intensities of the bands, which were quantitated by densitometry using Image Lab, normalizing to GAPDH levels. Data are represented as the mean ± SD (*n* = 3). *P < 0.05, **P < 0.01, ***P < 0.001

We next wondered how BMP-7 potentiates rSjp40’s inhibition on HSC activation. It has been demonstrated that phosphorylation of Smad1/5/8 is required for the inhibitory effect of BMP-7 on activation of HSCs [7, 10, 25]. We therefore examined whether phosphorylation of Smad1/5/8 is induced by rSjp40. The results showed that rSjp40 increased the level of p-Smad1/5/8 in con-siRNA group (t-test: *t*_(4)_ = 3.136, *P* = 0.0175). However, knockdown of BMP-7 markedly inhibited rSjp40-triggered phosphorylation of Smad1/5/8 in LX-2 cells (t-test: *t*_(4)_ = 6.767, *P* = 0.0012) (Fig. 2). These results suggest that rSjp40 inhibits activation of HSCs partially *via* BMP-7/Smad1/5/8-dependent mechanism.

### rSjp40 upregulates BMP-7 expression in LX-2 cells

**Fig. 3:**
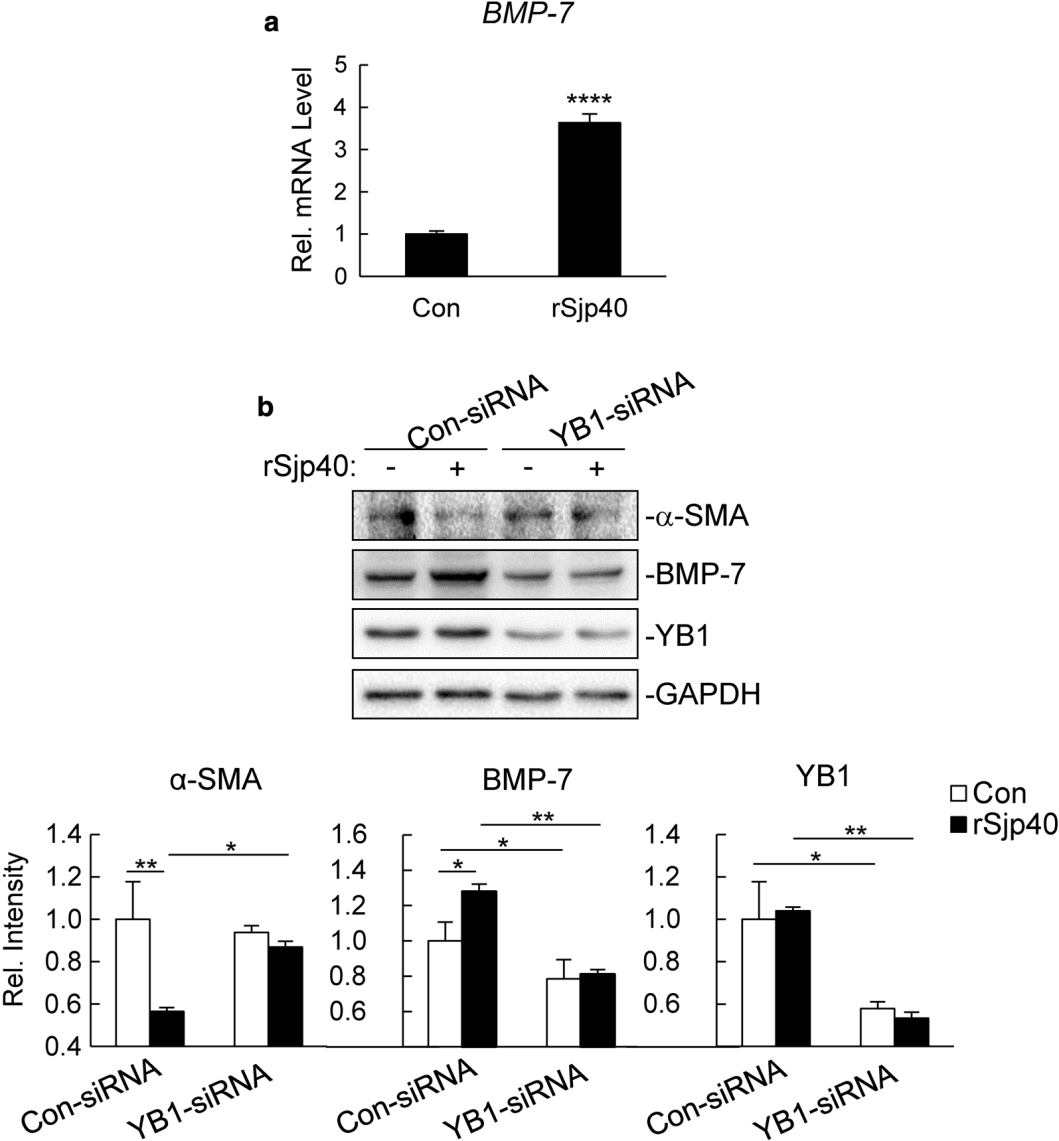
rSjp40-induced BMP-7 expression is dependent on YB1 in LX-2 cells. **a** LX-2 cells (2–3 × 10^5^) were left untreated or treated with rSjp40 (5 μg/ml) for 48 hours, and then total RNA was extracted for qPCR analysis. Data are represented as the mean ± SD (*n* = 3). ****P < 0.0001. **b** LX-2 cells (2–3 × 10^5^) were transfected with Con-siRNA or YB1-siRNA (300 pmol each). Forty-eight hours after transfection, cells were left untreated or treated with rSjp40 (5 μg/ml) for 48 hours before immunoblot analysis was performed with the indicated Abs. The bar graphs show the relative intensities of the bands, which were quantitated by densitometry using Image Lab, normalizing to GAPDH levels. Data are represented as the mean ± SD (*n* = 3). *P < 0.05, **P < 0.01

In the experiments above, we found that rSjp40 upregulated the protein expression of BMP-7 in con-siRNA LX-2 cells (t-test: *t*_(4)_ = 2.194, *P* = 0.0466) (t-test: *t*_(4)_ = 8.671, *P* = 0.0005) (Figs. 1, 2). Furthermore, quantitative real time PCR (qPCR) experiments showed that transcription of *BMP-7* gene was also significantly increased in rSjp40-treated LX-2 cells (t-test: *t*_(16)_ = 17.61, *P* < 0.0001) (Fig. 3a). These results suggest that rSjp40 upregulates BMP-7 expression at both mRNA and protein levels in LX-2 cells.

### rSjp40-induced BMP-7 expression is dependent on YB1 in LX-2 cells

Several studies suggest that YB1 is a transcriptional activator of BMP-7 [13, 14], thus, we explored whether rSjp40-induced BMP-7 expression is dependent on YB1 in LX-2 cells. RNAi experiments suggested that knockdown of YB1 decreased both basal and rSjp40-triggered expression of BMP-7 (t-test: *t*_(4)_ = 2.513, *P* = 0.0329) (t-test: *t*_(4)_ = 6.150, *P* = 0.0018) (Fig. 3b). Furthermore, the inhibitory effect of rSjp40 on α-SMA expression was partially reversed by YB1-siRNA (t-test: *t*_(4)_ = 3.648, *P* = 0.0109) (Fig. 3b). These results suggest that YB1 is a key regulator in rSjp40-induced BMP-7 expression and inhibition of activated HSCs.

### rSjp40 promotes the nuclear translocation of YB1 in LX-2 cells

**Fig. 4:**
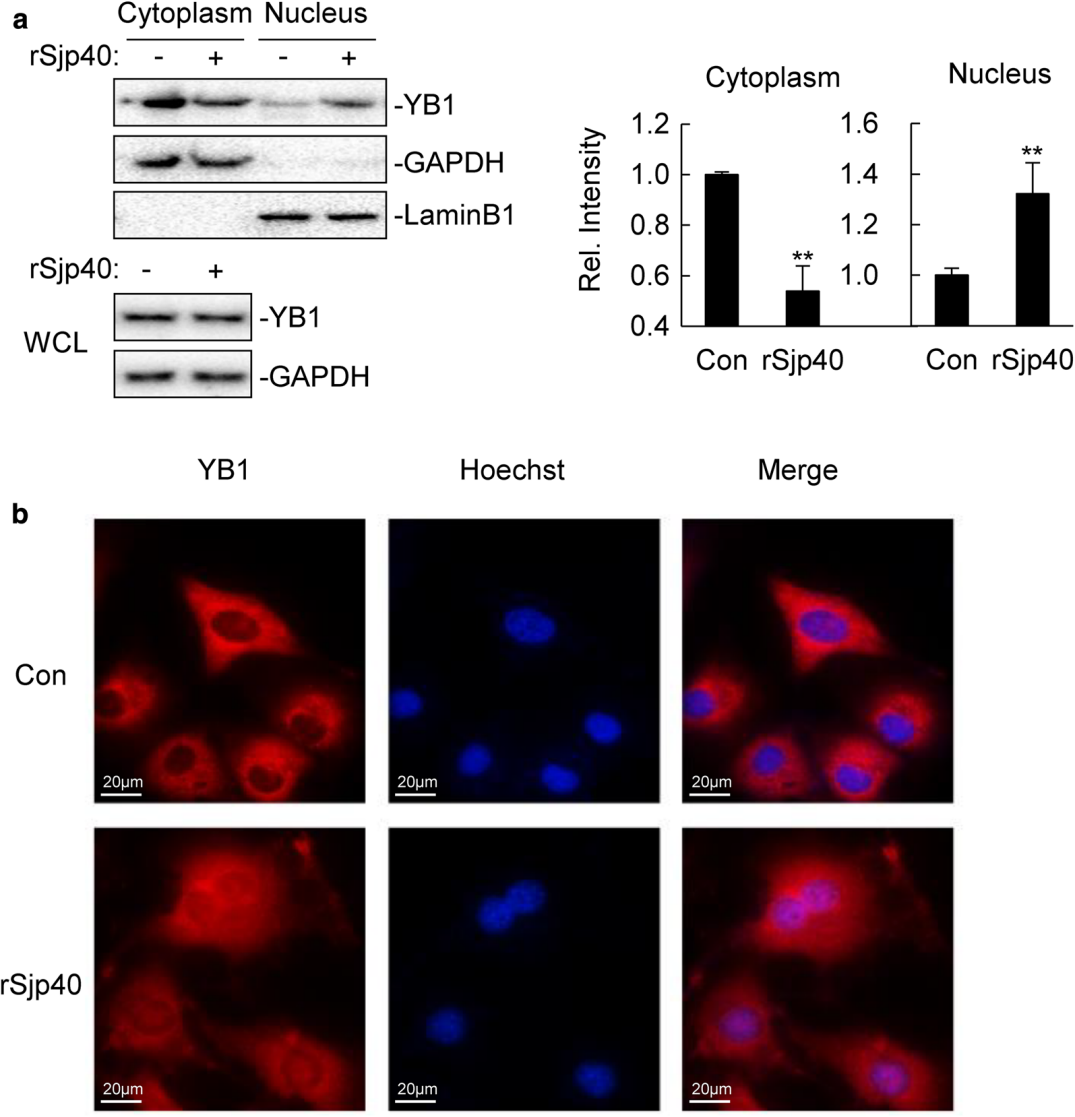
rSjp40 promotes the nuclear translocation of YB1 in LX-2 cells. **a** LX-2 cells (1–2 × 10^6^) were left untreated or treated with rSjp40 (5 μg/ml) for 48 hours. Then the cells fractionated, and the subcellular fractions, including cytoplasm and nucleus were equilibrated to equal volumes and analyzed by immunoblots with the indicated Abs. The bar graphs show the relative intensities of the bands, which were quantitated by densitometry using Image Lab, normalizing to GAPDH or LaminB1 levels. Data are represented as the mean ± SD (*n* = 3). **P < 0.01. **b** LX-2 cells (5–6 × 10^4^) were left untreated or treated with rSjp40 (5 μg/ml) for 48 hours. Then cells were fixed with 4% paraformaldehyde, and immunostaining was performed before observed by fluorescent microscopy. *Scale-bars*: **b**, 20 μm

We next investigated the mechanism of rSjp40 in the regulation of YB1. Since rSjp40 had little effect on total level of YB1 expression (Fig. 3b), we wondered whether rSjp40 is involved in YB1 nuclear translocation. It has been reported that YB1 regulated gene transcription in the nucleus only [14], therefore, nuclear translocation of YB1 is essential for it to exert its function. Cell fractionation experiments indicated that a major fraction of YB1 was located in the cytoplasm and only trace amount of YB1 was detected in the nucleus in untreated cells (Fig. 4a). Interestingly, upon rSjp40 treatment, the nuclear level of YB1 was increased (t-test: *t*_(4)_ = 4.553, *P* = 0.0052), whereas cytoplasmic level of YB1 was decreased (t-test: *t*_(4)_ = 6.503, *P* = 0.0014), with total level of YB1 unchanged (Fig. 4a). Fluorescent microscopy further confirmed that YB1 was translocated from the cytoplasm to the nucleus after rSjp40 treatment (Fig. 4b). These results suggest that rSjp40 promotes the nuclear translocation of YB1 in LX-2 cells.

## Discussion

In this study, we found that rSjp40 increased the phosphorylation level of Smad1/5/8 and decreased the expression of α-SMA in LX-2 cells. Interestingly, knockdown of BMP-7 inhibited rSjP40-triggered phosphorylation of Smad1/5/8 and downregulation of α-SMA. Based on the evidence that the anti-fibrotic actions of BMP-7 are due to its ability to counteract TGF-β1 signaling through increasing the phosphorylation level of Smad1/5/8 [7, 10, 25, 26], we therefore concluded that rSjp40 inhibits activation of HSCs partially through BMP-7/Smad1/5/8 signaling pathway. However, our results showed that the phosphorylation level of Smad1/5/8 in LX-2 cells transfected with BMP-7-siRNA and treated with rSjP40 was even lower than the LX-2 cells transfected with BMP-7-siRNA only (t-test: *t*_(4)_ = 3.276, *P* = 0.0153) (Fig. 2). It is possible that rSjp40 could directly inhibit the phosphorylation level of Smad1/5/8 *via* an unknown mechanism.

YB1 can be shuttled between the cytoplasm and the nucleus. Many compounds such as oxymatrine and Hsc025 can induce the nuclear translocation of YB1 [27, 28]. In contrast, TGF-β1 reduces the nuclear translocation of YB1 from the cytoplasm in Madin–Darby Canine Kidney (MDCK)-cells [14]. In this study, we found that the nuclear translocation of YB1 could also be induced by rSjP40 treatment. It is reported that p53 was required for the nuclear translocation of YB1, and the frequency of nuclear YB1 was dependent on the amount of p53 but not formation of p53/YB1 complex [29]. Our previous study showed that rSjp40 upregulated p53 expression in LX-2 cells [23]. Therefore, it is possible that rSjp40-induced nuclear translocation of YB1 is also dependent on p53.

According to previous reports, YB1 is also a negative regulator in liver fibrosis. One direct evidence is that overexpression of YB1 improved liver fibrosis induced by CCl_4_ in mice [30]. Adding to its mechanism, some studies showed that YB1 inhibited TGF-β1 signaling *via* acting on different targets [31, 32], while others reported that YB1 had the repressive effect on collagen type I gene transcription [33–35]. In our study, we showed that YB1 exerts anti-fibrotic action by increasing the level of BMP-7 expression in LX-2 cells.

## Conclusions

In conclusion, these present findings demonstrated that BMP-7 is required for rSjp40’s inhibition on HSC activation. Upon rSjp40 treatment, YB1 was translocated to the nucleus to promote BMP-7 expression, leading to phosphorylation of Smad1/5/8. Our studies provide a new clue to guide ongoing research into the anti-fibrosis of rSjp40.

## Data Availability

The data supporting the conclusions of this article are included within the article.

## Abbreviations

BMP: bone morphogenic protein
rSjp40: the recombinant *Schistosoma japonicum* protein p40
YB1: Y-Box protein-1
ECM: extracellular matrix
HSC: hepatic stellate cell
α-SMA: α-smooth muscle actin
TGF-β1: transforming growth factor-β1
